# MRI-based radiomics features uncover the micro-change of dorsal root ganglia lesion for patients with post-herpetic neuralgia

**DOI:** 10.3389/fneur.2023.1257648

**Published:** 2023-11-22

**Authors:** Xueqin Cao, Donglin Wen, Shangchen Yu, Hua Zheng, Gang Wu, Xianwei Zhang

**Affiliations:** ^1^Department of Anesthesiology, Hubei Key Laboratory of Geriatric Anesthesia and Perioperative Brain Health, Wuhan Clinical Research Center for Geriatric Anesthesia, Tongji Hospital, Tongji Medical College, Huazhong University of Science and Technology, Wuhan, China; ^2^Department of Radiology, Tongji Hospital, Tongji Medical College, Huazhong University of Science and Technology, Wuhan, China

**Keywords:** dorsal root ganglion, postherpetic neuralgia, magnetic resonance neurography, LASSO, algorithm, radiomics

## Abstract

**Objective:**

To create and authenticate MRI-based radiomic signatures to identify dorsal root ganglia (DRG) lesions in post-herpetic neuralgia (PHN) patients generalizable and interpretable.

**Method:**

This prospective diagnostic study was conducted between January 2021 and February 2022. Lesioned DRG in patients with PHN and normal DRG in age-, sex-, height-, and weight-matched healthy controls were selected for assessment and divided into two groups (8:2) randomly: training and testing sets. The least absolute shrinkage and selection operator algorithm was employed to generate feature signatures and construct a model, followed by the assessment of model efficacy using the area under the curve (AUC) of the receiver operating characteristic (ROC), as well as sensitivity and specificity metrics.

**Results:**

The present investigation involved 30 patients diagnosed with postherpetic neuralgia (PHN), consisting of 18 males and 12 females (mean age 60.70 ± 10.18 years), as well as 30 healthy controls, comprising 18 males and 12 females (mean age 58.13 ± 10.54 years). A total of 98 DRG were randomly divided into two groups (8:2), namely a training set (*n* = 78) and a testing set (*n* = 20). Five radiomic features were chosen to construct the models. In the training dataset, the area under the curve (AUC) was 0.847, while the sensitivity and specificity were 71.79 and 97.44%, respectively. In the test dataset, the AUC was 0.87, and the sensitivity and specificity were 80.00 and 100.00%, respectively.

**Conclusion:**

An MRI-based radiomic signatures model has the capacity to uncover the micro-change of damaged DRG in individuals afflicted with postherpetic neuralgia.

## 1 Introduction

Post-herpetic neuralgia (PHN), which is characterized by pain that endures for more than 1 month after the resolution of herpetic skin lesions or for more than 3 months following herpes zoster (HZ), represents the most prevalent complication of HZ infection (1). The duration of neuropathic pain ranges from several months to a lifetime, impacting 5–20% of individuals with HZ (2).

Dorsal root ganglia (DRG) is a critical lesioned structure for PHN (2). Recently, DRG-based targeted therapy, such as radiofrequency modulation and drug injection, has emerged as a safe and potentially effective treatment for PHN (3, 4). At present, the location of the target DRG is mainly based on the distribution of the herpetic skin lesions and subjective feelings of the patients during treatment, since *in vivo* examination of DRG in patients with PHN is challenging (5–7). However, location of the lesioned DRG may be inaccurate owing to increased age and communication difficulties, thereby affecting the treatment outcomes. Moreover, the number of DRG targets selected for treatment has been a considerable focus of discussion among clinicians. The number of DRG to be selected for treatment, primary target DRG, and whether DRG adjacent to the lesion segments need to be treated simultaneously are other treatment considerations, which have always been the focus of debate among clinical experts (5). Furthermore, the diagnosis and location of the target DRG for patients with zoster sine herpete are very challenging (8). So, identifying the lesioned DRG in patients with PHN through non-invasive objective imaging examination is warranted.

Our previous study found DRG on the lesioned side to be swollen during PHN, but there was a tendency for a reduction in the volume of DRG with the prolongation of the disease course (unpublished). This implies that the identification of DRGs through macroscopic changes poses a challenge. Radiomics is a nascent discipline in quantitative imaging that employs sophisticated mathematical algorithms to extract high-throughput quantitative features from medical images, thereby providing an objective and quantitative depiction of tissue characteristics that are imperceptible to the human eye (9, 10). By capturing tissue and lesion characteristics, radiomics offers a potential solution for clinical problem-solving, including identification and prediction (11).

Over the last 10 years, radiomics has gained significant traction in cancer research. Notably, radiomic features have demonstrated potential in prognosticating treatment response, distinguishing between benign and malignant tumors, and evaluating cancer genetics across various cancer types (12, 13). For instance, Hugo et al. employed radiomics to identify a common prognostic phenotype in head-and-neck and lung cancer (14). Additionally, radiomics has been applied in the investigation of other medical conditions, including metabolic syndrome, Parkinson’s disease, and cardiac sarcoidosis (15, 16). In their study, Shi and colleagues discovered a significant correlation between Run Entropy and the primary metabolic outcomes as well as the long-term prognosis of individuals diagnosed with metabolic syndrome (17). Ren and colleagues developed a model utilizing radiomic features to differentiate between PDs and non-PDs (18). Osamu and colleagues identified that Short Run Low Gray Level Emphasis exhibited high specificity in diagnosing nodular cardiomyopathy (16).

Here, based on the region of interest (ROI) for lesioned and control DRG, we aimed to extract high-throughput radiomics features and construct a predictive model to distinguish lesioned DRG from normal DRG. We aimed to (1) provide a group of radiomics features for the identification of lesioned DRG *in vivo* and (2) postulate an anatomical basis for individualized treatment of PHN.

## 2 Materials and methods

The present investigation was executed in compliance with the Declaration of Helsinki and the research plan was sanctioned by the institutional Ethics Committee (2021S084). The study is duly registered at ClinicalTrials.gov. Prior to their involvement in the study, all participants provided written informed consent.

### 2.1 Study design and population

**Figure 1:**
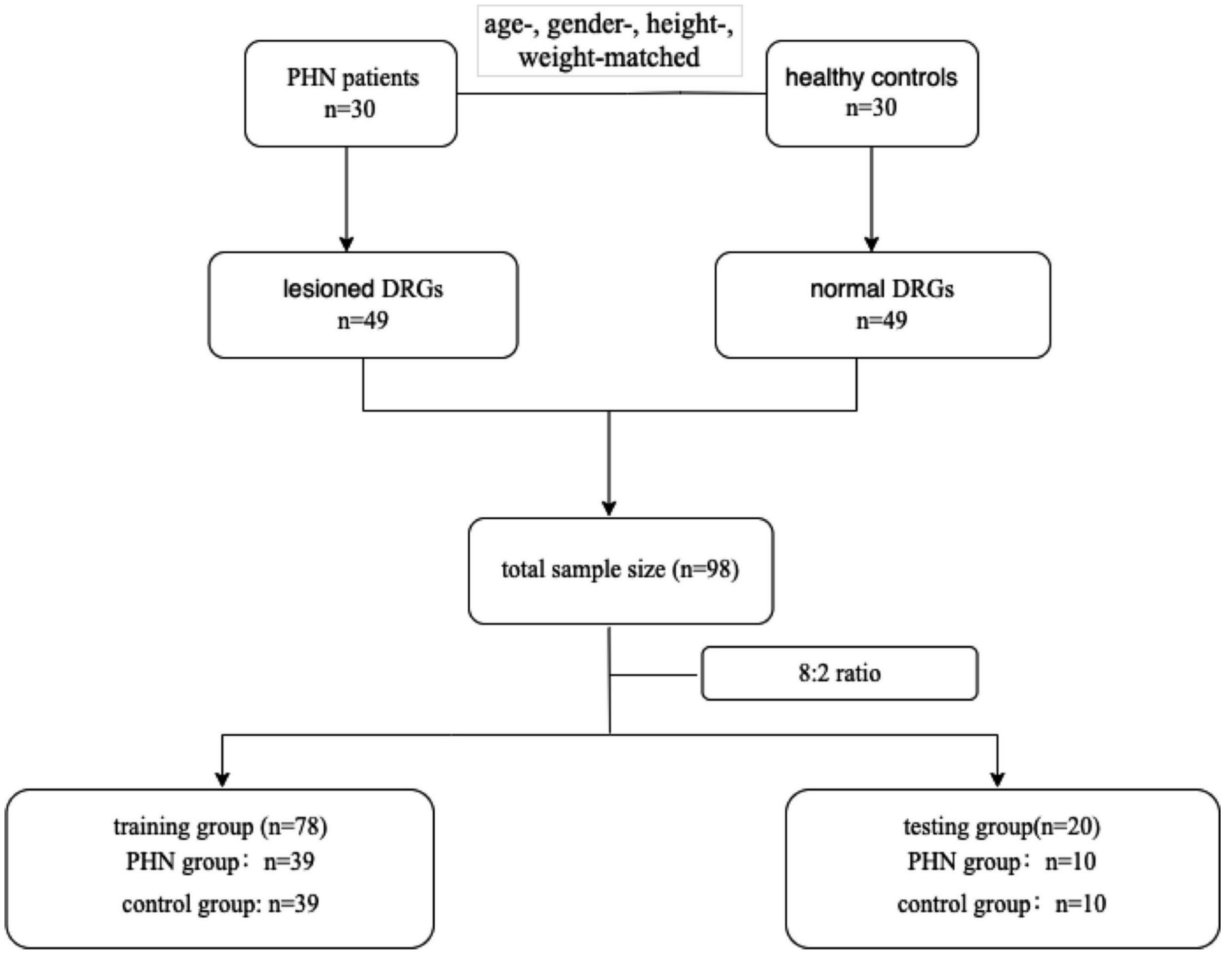
Flowchart of participants enrolment.

This was a prospective, diagnostic study. Thirty patients with PHN in the thoracic segments admitted to our hospital between January 2021 and February 2022 were recruited. The patient inclusion criteria were age > 18 years and pain lasting for at least 1 month after lesion crusting. The study employed exclusion criteria that encompassed severe systemic, metabolic, or neurological diseases that may be associated with peripheral polyneuropathies, multiple myeloma, diabetes mellitus, or thyroid disease, as well as a history of psychiatric diseases, chronic pain, or substance abuse, inability to complete magnetic resonance neurography (MRN) scans, and a history of thoracic surgery and pain. Furthermore, the study included 30 healthy controls who were matched in terms of age, sex, height, and weight. A flowchart of participant enrolment is shown in Figure 1.

### 2.2 Clinical locating the lesion dermatomes

The dermatome lesions of all patients were identified based on the cutaneous nerve distribution schematic in the trunk (Atlas of Human Anatomy, 3rd edition, Frank H. Netter) by two pain specialists with over a decade of clinical experience (Authors X and Y).

### 2.3 Imaging scan

**Figure 2:**
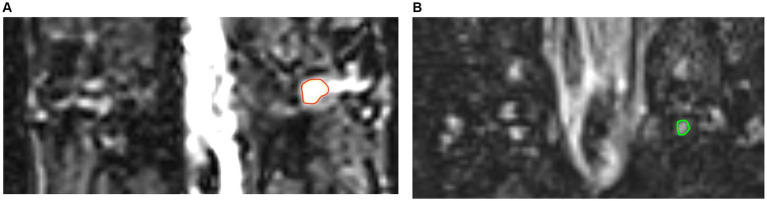
The images of lesioned and normal DRG. **(A)** The image in red ring shows lesioned DRG of PHN patient. **(B)** The image in green ring shows normal DRG in healthy control.

A 3.0 Tesla MRI clinical scanner (Magnetom Skyra; Siemens Healthcare, Germany) was used. All the patients underwent magnetic resonance imaging (MRI) before treatment initiation. All the participants laid in supine position on the scanner bed with their thorax immobilized in a tight-fitting thoracic coil, and underwent MRN as follows: Three-dimensional T2-weighted sampling perfection with application-optimized contrasts using different flip-angle evolution short-tau inversion-recovery (3-D SPACE STIR) sequence of the spine was used for DRG and spinal nerve imaging: coronal plane; repetition time/echo time, 3,000/178 ms; inversion time, 220 ms; field of view, 305 × 240 mm^2^; matrix size, 320 × 320; slice thickness, 1.0 mm; slice number, 60; no gap; voxel size, 1.0 × 1.0 × 1.0 mm^3^; 50% phase over-sampling; and acquisition time, 8 min 35 s; imaging all bilateral DRG in the thoracic spine. All the bilateral DRG in the thoracic spine were also scanned in the healthy controls. Subsequently, 49 lesioned DRG from 30 patients were chosen for evaluation based on dermatome lesions, while an equivalent number of 49 normal DRG from the corresponding segments of 30 healthy controls were also chosen for comparison. Figure 2 shows images of the lesioned and normal DRG.

### 2.4 Image segmentation

A radiologist (Author Z) with 4 years of experience performed manual segmentation of two-dimensional ROI on the 3-D SPACE STIR coronal MR image of the DRG utilizing the 3D-slicer software (version 5.0.3).^1^ The ROI mask was selected as the DRGs in the lesioned segments of patients with PHN and in the corresponding segments of healthy controls. The DRG margins were delineated by covering the largest cross-section of the DRG. Intra-observer agreement assessment was conducted by the same radiologist performing a second ROI manual segmentation after a two-week interval. Another radiologist (Author W) with 13 years of experience independently performed ROI segmentation using the same approach to assess inter-observer reliability. All the ROIs were saved locally for further analysis. The clinical data was concealed from both radiologists during the evaluation process. The reliability of their observations was assessed through the use of intra-and inter-observer correlation coefficients (ICCs), with values exceeding 0.75 indicating a high level of agreement.

### 2.5 Image normalization and radiomic feature extraction

Texture recognition was improved by applying several preprocessing methods. All the MR images in this study were scanned by the same radiologist using the same MRI scanner with the same parameters to ensure homogeneity of the MR Images. The voxel sizes of all images were standardized to 1 × 1 × 1 mm^3^ through resampling.

**Figure 3:**
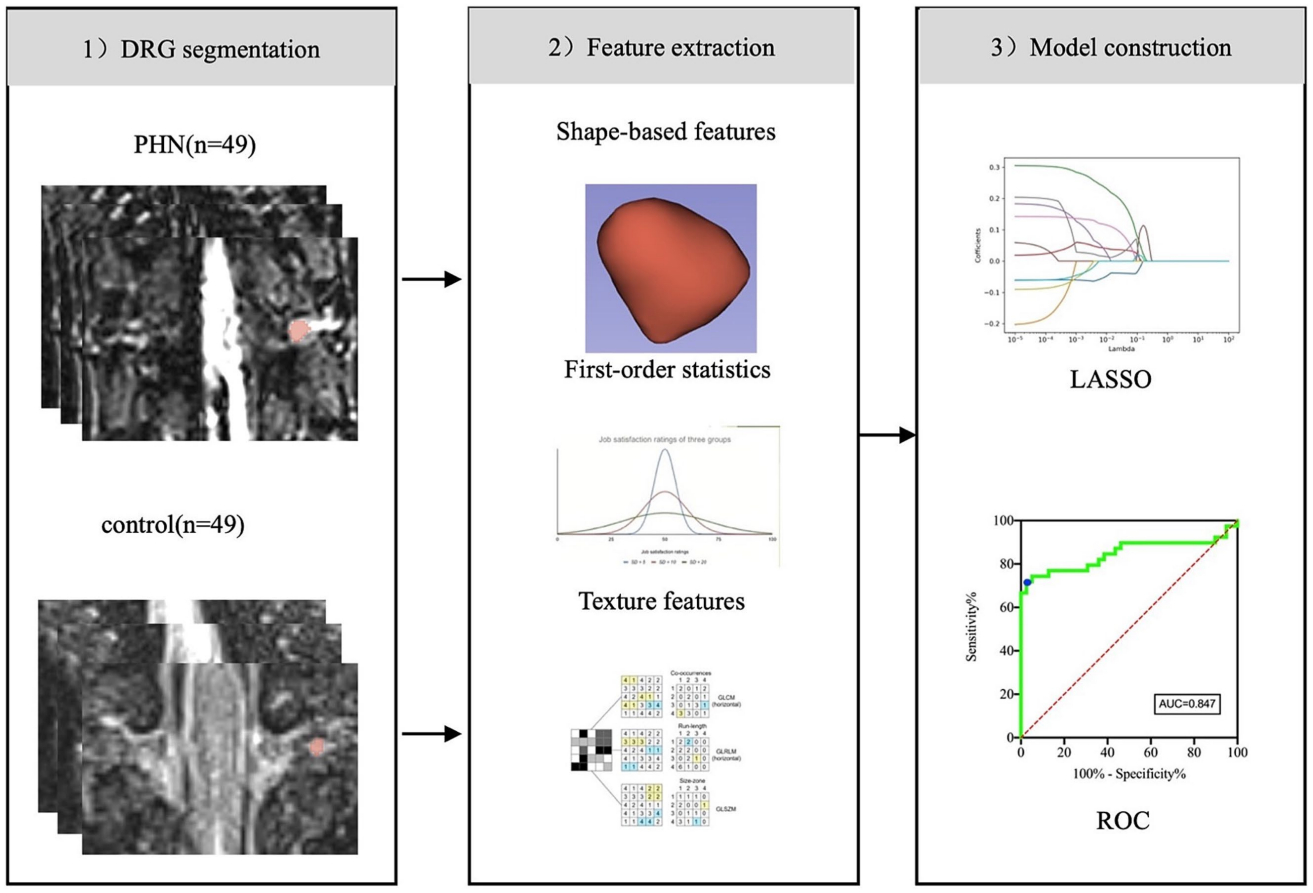
The radiomics workflow.

The radiomics workflow is shown in Figure 3. The PyRadiomics (version 3.0.1; Computational Imaging and Bioinformatics Lab, Harvard Medical School) package in the 3D-slicer software was utilized to perform image feature extraction on all the ROIs. This process resulted in the extraction of high-throughput radiomics features, which included first-order statistics, shape-based features, gray-level co-occurrence matrix (GLCM), gray-level run length matrix (GLRLM), gray-level size zone matrix (GLSZM), neighboring gray-tone difference matrix (NGTDM), and gray-level dependence matrix (GLDM).

### 2.6 Feature selection and signatures establishment

The current investigation employed the LASSO regression analysis technique to ascertain the most relevant characteristics in the training dataset. LASSO has been shown to be a proficient technique for generating sparse solutions in the context of high-dimensional data. Through the integration of a penalty function and linear regression, the coefficients of candidate features with negligible impact are reduced to zero, while those with non-zero coefficients are retained. The LASSO algorithm was employed for the purpose of feature selection and signature establishment in this investigation, encompassing both training and validation phases.

The LASSO algorithm (anaconda/spider, version: 2019.03) was employed for the purpose of feature selection and signature establishment in this investigation. The training and validation cohorts were partitioned into two datasets at an 8:2 ratio, with DRGs being randomly allocated to either the training or validation cohorts. The training and testing cohorts were comprised of 78 and 20 DRGs, respectively.

mRMR algorithm (Max-Relevance and Min-Redundancy) (anaconda/spider, version: 2019.03) was used to eliminate redundant and irrelevant features. Finally, the top 15 features were selected. We then employed LASSO to reduce some feature coefficients to zero, ultimately retaining only the five most significant coefficient features to construct the LASSO regression model. By combining these features, a radiomics signature was established for each DRG.

### 2.7 Evaluation parameters

In order to assess the efficacy of the models, a variety of performance metrics were employed, including the area under the receiver operating characteristic (ROC) curve (AUC), sensitivity, and specificity. The ROC curve indicated that the optimal cutoff risk score was situated closest to the upper left corner, resulting in heightened sensitivity and specificity, as well as a reduction in false positives and negatives. The maximum Youden index (YI = sensitivity + specificity-1) was utilized to determine the optimal cutoff risk score.

### 2.8 Statistical analysis

The study collected demographic information of participants in the form of numerical data, mean ± standard deviation, or median (interquartile range) for continuous and categorical variables. Statistical analyses were conducted using Prism software (version 9.0), and AUC curves were plotted. Continuous variables were assessed using independent *t*-tests, paired *t*-tests, or Wilcoxon tests, while categorical variables were analyzed using the chi-squared test or Fisher’s exact test. LASSO regression analysis was performed using Python software (version 3.11.4). All statistical tests were two-sided, and statistical significance was determined.

## 3 Results

### 3.1 Demographic and clinical characteristics

The research cohort consisted of 49 lesioned DRGs obtained from 30 individuals diagnosed with PHN. Of these individuals, 18 were male and 12 were female, with ages ranging from 36 to 82 years and a mean age of 60.7 years. The average duration of the disease course for these patients was 2.65 (1.5–6.0) months. The control group comprised 49 DRGs obtained from 30 healthy individuals, matched for age, sex, weight, and height. The control group consisted of 18 male and 12 female individuals, with ages ranging from 36 to 78 years and a mean age of 58.13 years. Table 1 presents the demographic and clinical characteristics of the participants.

**Table 1:** Characteristics of postherpetic neuralgia (PHN) patients and healthy control group.

Characteristic	PHN cases (*n* = 30)	Healthy controls (*n* = 30)	*p*
Mean age, years	60.70 ± 10.18	58.13 ± 10.54	0.341
Gender, n; female/male	12/18	12/18	
Mean height-cm	163.4 ± 7.29	164.0 ± 7.22	0.598
Mean weight-kg	61.1 ± 11.45	62.9 ± 7.91	0.468
Total lesion dermatomes	49		
Mean lesion dermatome (per patient)	1.63 ± 0.49	—	

Continuous data are presented as means ± SDs; Categorical data are numerators.

### 3.2 Radiomic feature selection, and radiomic signature establishment

**Figure 4:**
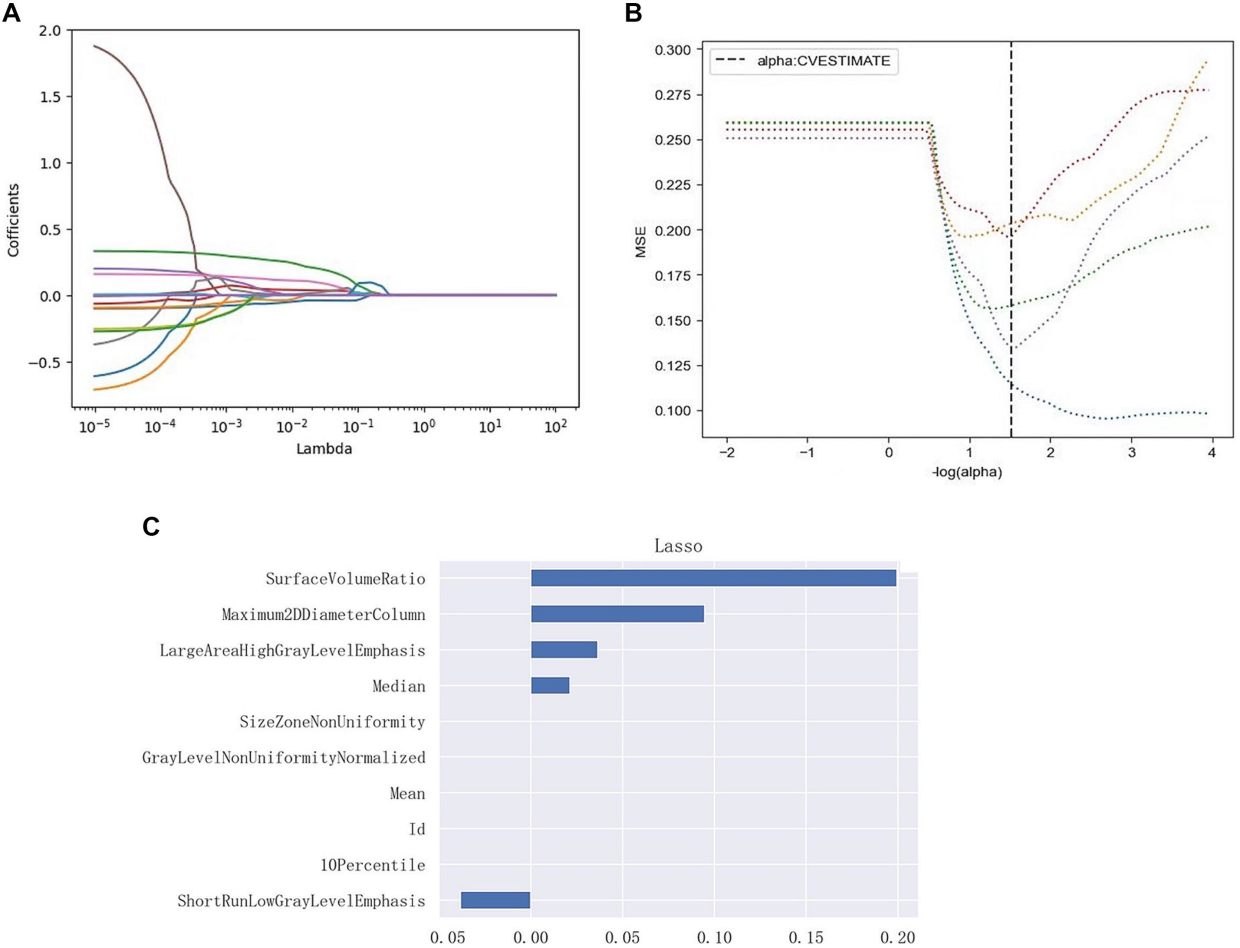
Radiomic feature selection, and radiomic signature establishment using LASSO. **(A)** Log lambda and coefficients plot. As lambda increases, the coefficients shrink toward zero, indicating the features’ decreasing importance in predicting the target variable. **(B)** Log lambda and mean squared error (MSE) plot. The dashed black line indicates the optimal regularization strength chosen by cross-validation to minimize the MSE on the test data. **(C)** Lasso Regression Coefficients Chart for each radiomics signature. The retained non-zero coefficient features are plotted on the *y*-axis and their coefficients in the LASSO Cox analysis are plotted on the *x*-axis. Its shows the impact of the radiomics features on the target variable.

Intra-and inter observer reliabilities were evaluated using correlation coefficients, and the ICCs were 0.92 and 0.85, respectively, indicating good stability. A total of 103 features were retained for the analysis. To construct the model, three plots were generated: log lambda and coefficient plots, log lambda and mean squared error (MSE) plots, and the impact of each feature on the target variable was assessed (Figure 4). From the training dataset, the five PHN-related features with the highest predictive capability were identified, comprising of two shape features, one first-order feature, and two texture features (one GLRLM and one GLSZM feature).

### 3.3 Efficacy evaluation of the LASSO regression model

**Figure 5:**
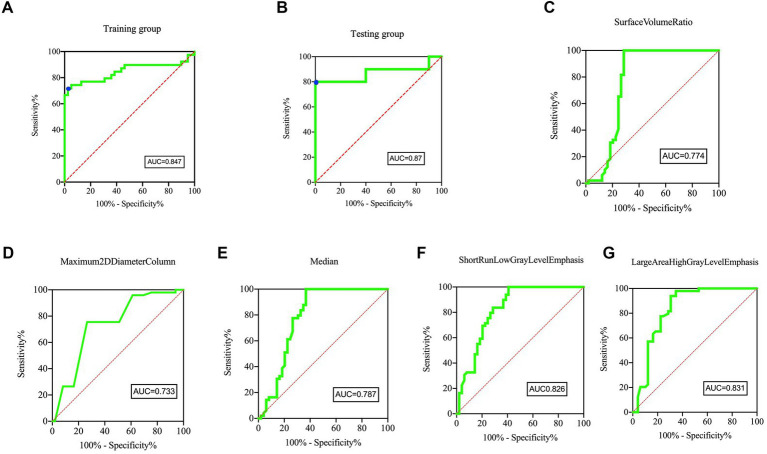
ROC curves of the Model and five individual radiomics features. The ROC curves of model in panel **(A)** Training group and **(B)** Testing group, showing the feature signature performed best. For the training group, the AUC was 0.847 [95% confidence interval, CI, 0.750–0.944]. The sensitivity and specificity were 71.79 and 97.44% at the optimal cut-off value (0.423), respectively (blue point). For the testing group, the AUC was 0.87 [95% confidence interval, CI, 0.687–1]. The sensitivity and specificity were 80.00 and 100.00% at the optimal cut-off value (0.567), respectively (blue point). The AUC of five individual radiomic features were 0.774 **(C)**, 0.733 **(D)**, 0.787 **(E)**, 0.826 **(F)**, 0.831 **(G)**, respectively.

Figure 5 displays the ROC plots of the model, which offer a visual representation of its performance and reliability in distinguishing lesioned DRGs in PHN from normal DRGs. The AUC for the training dataset was 0.847 (95% CI, 0.750–0.944), with a sensitivity of 71.79% and specificity of 97.44% at an optimal cutoff value of 0.423. The AUC for the test dataset was 0.87 (95% CI, 0.687–1), with a sensitivity of 80% and specificity of 100% at an optimal cutoff value of 0.567.

### 3.4 Difference in the five radiomics features between lesioned DRGs in patients with PHN and normal DRGs in healthy control

**Figure 6:**
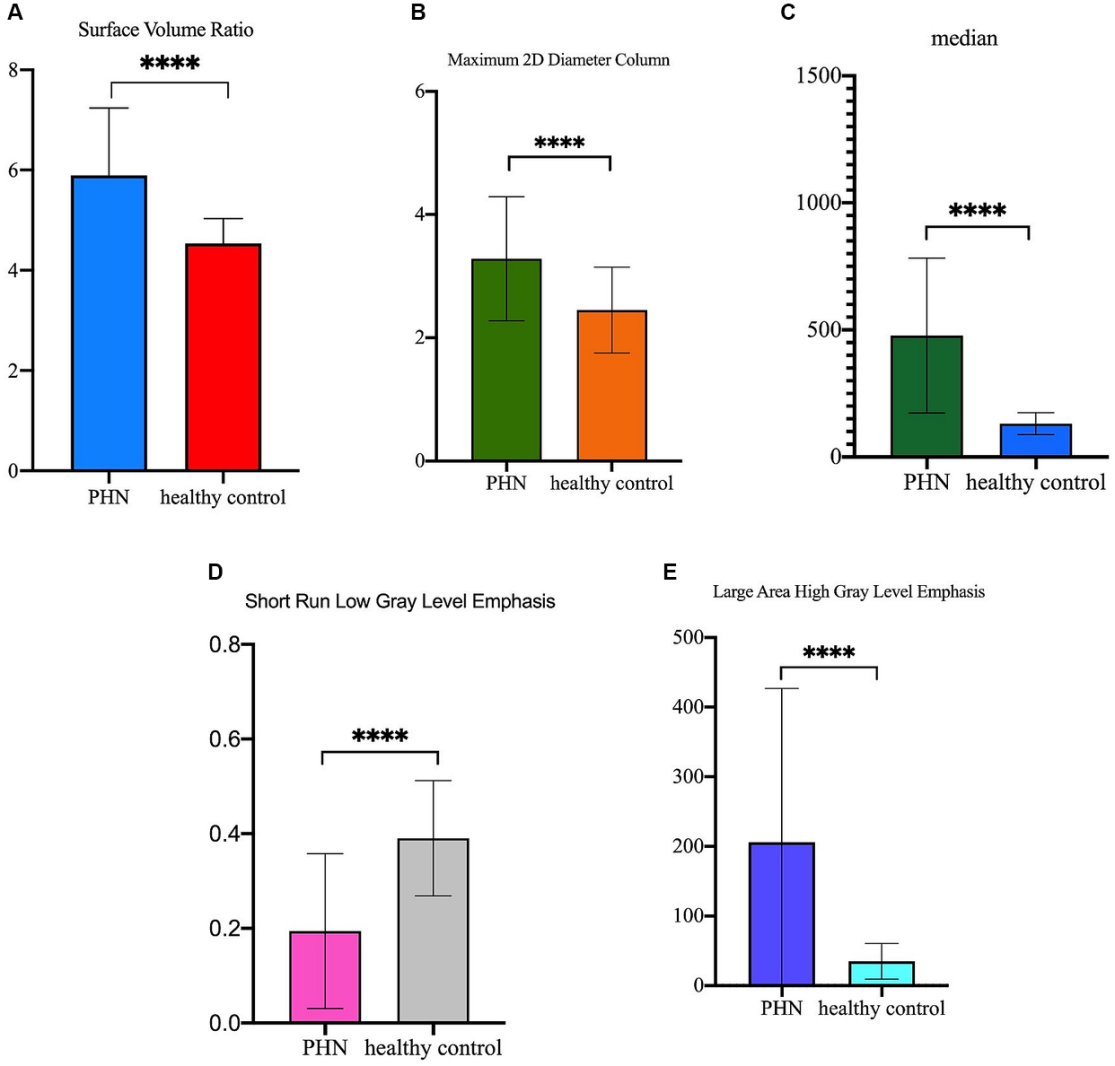
The difference of the five radiomics features between lesioned DRGs in PHN patients and normal DRGs in healthy control. **(A)** Surface Volume Ratio of lesioned DRG was significantly more than DRG in healthy control (5.90 ± 1.34 vs. 4.54 ± 0.50, *p* ≤ 0.0001); **(B)** Maximum 2D Diameter Column of lesioned DRG was significantly more than DRG in healthy control (3.281 ± 1.008 vs. 2.447 ± 0.696, *p* ≤ 0.0001); **(C)** Median of lesioned DRG was significantly more than DRG in healthy control (477.4 ± 304.4 vs. 131.5 ± 43.08, *p* ≤ 0.0001); **(D)** Short Run Low Gray Level Emphasis of lesioned DRG was significantly less than DRG in healthy control (0.19 ± 0.16 vs. 0.39 ± 0.12, *p* ≤ 0.0001); **(E)** Large Area High Gray Level Emphasis of lesioned DRG was significantly more than DRG in healthy control (205.8 ± 221.0 vs. 34.82 ± 25.72, *p* ≤ 0.0001).

As showed in Figure 6, the Surface Volume Ratio, Maximum 2D Diameter Column, Median and Large Area High Gray Level Emphasis of the lesioned DRG were significantly more than the DRG in healthy controls (5.90 ± 1.34 vs. 4.54 ± 0.50, 3.281 ± 1.008 vs. 2.447 ± 0.696, 477.4 ± 304.4 vs. 131.5 ± 43.08, and 205.8 ± 221.0 vs. 34.82 ± 25.72, respectively, *p* ≤ 0.0001). The Short Run Low Gray Level Emphasis of lesioned DRG were significantly less than the DRG in healthy controls (0.19 ± 0.16 vs. 0.39 ± 0.12, *p* ≤ 0.0001).

## 4 Discussion

The objective of this investigation was to examine the radiomic characteristics that differentiate lesioned DRG in individuals with PHN from healthy DRGs. A total of forty-five radiomics features were observed to exhibit differences between lesioned DRG and normal DRG. Among these features, five were utilized to develop diagnostic models for the identification of lesioned DRG. To our knowledge, this is the initial study to construct a radiomics-based diagnostic model for a lesioned DRG in PHN. The findings indicate that the model exhibited a higher AUC than the five individual radiomics features, suggesting its superiority over the latter.

Medical imaging is widely employed in clinical practice worldwide for the purposes of diagnosing, guiding treatment, and monitoring diseases, due to its noninvasive and timely assessment of human tissue characteristics (19). The morphological characteristics of conventional MR images are often employed as the basis for differential diagnosis. Nonetheless, the diagnostic efficacy of these features is contingent upon the radiologist’s proficiency and expertise in interpreting MR images. In contrast, radiomic features capture a substantial amount of information and exhibit significant potential for personalized clinical applications, distinguishing them from traditional morphological features.

Many studies have demonstrated different radiomics features to be significantly correlated with different genetic changes, clinical features, and important prognostic markers (20). Hempel et al. found that normalized mean kurtosis was significantly reduced in tumors with *ATRX* mutations and was a potential *in vivo* biomarker for the diagnosis of glioma (12). In our study, the five radiomics features constructed from the model were Surface Volume Ratio, Maximum 2D Diameter Column, Median, Short Run Low Gray Level Emphasis, and Large Area High Gray Level Emphasis. The model’s area under the curve was determined to be 0.847 and 0.87 for the training and test datasets, respectively. As such, the five radiomics features may be considered as radiomics signatures associated with PHN.

The Surface Volume Ratio reflects the compactness of the ROI, with lower values indicating a more compact, spherical shape (10). The Maximum 2D Diameter Column measured the maximum two-dimensional two-point distance in the coronal plane. This study reveals that the Surface Volume Ratio of lesioned DRG in individuals with PHN exhibited a statistically significant increase compared to the normal DRG in healthy controls. This finding suggests a significant alteration in DRG morphology among patients with PHN. The Maximum 2D Diameter Column was significantly larger than that of the normal DRG in healthy controls, implying a larger volume of the lesioned DRG. The findings of our study demonstrate a significant increase in the volume of lesioned dorsal root ganglia (DRG) among patients with postherpetic neuralgia (PHN), which is in line with the results of our prior investigation (unpublished). Furthermore, the Median, a first-order feature, reflects the average gray level, which was significantly increased in the lesioned DRGs of patients with PHN. Inflammatory responses are critical pathophysiological processes in PHN (21). In the postmortem analysis of one patient with PHN for 5 weeks, inflammatory responses were observed in the spinal dorsal horn manifesting as macrophage and lymphocyte infiltration, corresponding to the MRI T2-weighted hyperintensity (22). Recent research has indicated that the upregulation of both tumor necrosis factor-alpha (TNF-α) and interleukin (IL)-1β in the DRG is a persistent occurrence in cases of neuropathic pain (23). Thus, our report suggests that inflammatory responses persist in the DRG of patients with PHN.

Texture analysis involves computational methods that quantify tissue heterogeneity (10). Previous studies have suggested that evaluating intra-tumoral heterogeneity by quantifying the texture features of tumor PET images can distinguish between benign and malignant tumors and predict tumor response to the treatment (24, 25). The radiomics signatures extracted in this study included two textures: Short Run Low Gray Level Emphasis and Large Area High Gray Level Emphasis.

Short Run Low Gray Level Emphasis, a GLRLM feature, measures the degree of the regional distribution of low-intensity voxels with a short run length (related to fine texture) (10) and reflects the degree of texture fineness (25). The Large Area High Gray Level Emphasis, a measure of the combined distribution of larger zones with higher gray-level values in an image, is a property of the GLSZM. A higher Large Area High Gray Level Emphasis value indicates a greater heterogeneity of tissue texture (26). The current investigation revealed that the Short Run Low Gray Level Emphasis of lesioned DRGs in individuals with PHN was notably lower in comparison to healthy controls, indicating a coarser texture of the lesioned DRGs. Furthermore, the Large Area High Gray Level Emphasis of lesioned DRGs in individuals with PHN was significantly higher than that of healthy controls, indicating a greater degree of tissue heterogeneity in DRGs of individuals with PHN.

The dorsal root ganglia (DRG) comprise diverse populations of sensory and satellite glial cells with varying neurochemical profiles. DRG stress can induce notable changes in cell size (swelling/atrophy) and in distinct subcellular structures (e.g., mitochondria, endoplasmic reticulum, and nucleus) of neurons and/or satellite glial cells (27). Consequently, leveraging the DRG’s heterogeneity and coarse texture via radiomics could facilitate the investigation of postherpetic neuralgia (PHN) at the cellular and molecular levels.

Based on the aforementioned analysis, the model may be deemed a valuable biomarker for detecting lesioned DRG in PHN. Furthermore, in contrast to a solitary radiomics feature, the radiomics signature comprising the aforementioned five features presents a superior advantage in identifying the lesioned DRG, thereby exhibiting significant potential for personalized clinical applications.

Nonetheless, our study has certain limitations: (1) the selection of lesion DRG was based on dermatome lesions, Errors are inevitable; (2) a relatively limited sample size due to the singular-center nature of the study; (3) the absence of external validation as DRG imaging detection has yet to be implemented in clinical practice. To substantiate our results, a multicenter study with a more expansive sample size is recommended for future research.

In summary, a model based on radiomic signatures for identifying lesioned DRGs in patients with PHN was constructed for the first time, which was superiority over five individual radiomics features. The findings of this study could offer direction for the non-intrusive identification of damaged dorsal root ganglia and establish a visual foundation for personalized interventional management of postherpetic neuralgia.

## Data availability statement

The original contributions presented in the study are included in the article/Supplementary material, further inquiries can be directed to the corresponding authors.

## Ethics statement

The studies involving humans were approved by the Ethics Committee of Tongji Medical College, Huazhong University of Science and Technology, Wuhan, China. The studies were conducted in accordance with the local legislation and institutional requirements. The participants provided their written informed consent to participate in this study.

## Author contributions

XC: Conceptualization, Writing – original draft, Writing – review & editing, Data curation, Formal analysis, Investigation, Project administration, Software, Visualization. DW: Data curation, Formal analysis, Investigation, Software, Visualization, Writing – original draft, Writing – review & editing. SY: Data curation, Writing – original draft, Writing – review & editing, Validation. HZ: Validation, Writing – original draft, Writing – review & editing, Methodology. GW: Methodology, Writing – original draft, Writing – review & editing, Formal analysis, Resources, Visualization. XZ: Writing – original draft, Writing – review & editing, Conceptualization, Supervision.

## Footnotes

^1^ http://www.slicer.org/
